# Damping under Varying Frequencies, Mechanical Properties, and Failure Modes of Flax/Polypropylene Composites

**DOI:** 10.3390/polym15041042

**Published:** 2023-02-19

**Authors:** Md Zillur Rahman, Huaizhong Xu

**Affiliations:** 1 Department of Mechanical Engineering, Ahsanullah University of Science and Technology (AUST), Dhaka 1208, Bangladesh; 2 Department of Biobased Materials Science, Kyoto Institute of Technology (KIT), Matsugasaki Hashikamicho 1, Sakyoku, Kyoto 606-8585, Japan

**Keywords:** composite materials, flax fibre composites, mechanical properties, failure modes, damping, loss factor

## Abstract

This work investigates the effects of fibre content, fibre orientation, and frequency on the dynamic behaviour of flax fibre-reinforced polypropylene composites (FFPCs) to improve understanding of the parameters affecting vibration damping in FFPCs. The effects of fibre content and fibre orientation on the mechanical performances of FFPCs, along with fracture characteristics, are also investigated in this study. Laminates of various fibre contents and orientations were manufactured by a vacuum bagging process, and their dynamic and static properties were then obtained using dynamic (dynamic mechanical analysis (DMA) to frequencies of 100 Hz) and various mechanical (tensile and flexural) analyses, respectively. The findings suggest that of all the parameters, fibre orientation has the most significant impact on the damping, and the maximum loss factor (i.e., 4.3–5.5%) is obtained for 45° and 60° fibre orientations. However, there is no significant difference in loss factors among the composites with different fibre contents. The loss factors lie mainly in the range of 4–5.5%, irrespective of the fibre volume fraction, fibre orientation, and frequency. A significant improvement (281 to 953%) in damping is feasible in flax fibre/polypropylene composites relative to more widespread glass/epoxy composites. The mechanical properties of composites are also strongly affected by fibre orientation with respect to the loading direction; for example, the tensile modulus decreases from 20 GPa to 3.45 GPa at an off-axis angle of 30° for a fibre volume fraction of 0.40. The largest mechanical properties (tensile and flexural) are found in the case of 0° fibre orientation. For composites with fibre volume fractions in the range 0.31–0.50, tensile moduli are in the range 16–21 GPa, and tensile strengths are in the range 125–173 MPa, while flexural moduli and strengths are in the ranges 12–15 GPa and 96–121 MPa, respectively, making them suitable for structural applications. The obtained results also suggest that flax fibre composites are comparable to glass fibre composites, especially in terms of specific stiffness. The ESEM analysis confirms the tensile failures of specimens due to fibre debonding, fibre pull-out and breakage, matrix cracking, and inadequate fibre/matrix adhesion. The outcomes from this study indicate that flax fibre-reinforced composite could be a commercially viable material for applications in which noise and vibration are significant issues and where a significant amount of damping is required with a combination of high stiffness and low weight.

## 1. Introduction

The use of plant fibres as reinforcements is growing increasingly for various structural applications (e.g., vehicle chassis, aircraft airframes, bridges, ballistics, slabs, hand railings, and parquet flooring) due to their superior mechanical properties (high stiffness and strength to weight ratio), promising durability performance (corrosion resistance), ease of processing, non-toxic and eco-friendly nature, and low cost. Apart from their sustainability, low weight, wide availability, and biodegradability, plant fibres have high fracture toughness and high flexibility [1]. However, next to the demand for mechanical properties in various engineering applications, composite materials are often subjected to vibration, which leads to further demands for good dynamic behavior (e.g., high-vibration damping). The composite structures can be exposed to dynamic loadings due to fast-moving traffic, collisions, explosive blasts, wind-driven objects, ballistic projectiles, earthquakes, and machine vibrations. Despite the fact that there has been little research in this area, experimental findings from prior literature [2,3,4,5,6,7,8,9,10,11,12,13] lead to some indications concerning the dynamic properties of plant fibre-based composites utilizing dynamic mechanical analysis (DMA). The loss factors of flax-, glass-, and carbon-epoxy composites were studied by Duc et al. [2,3]. They revealed that flax fibre-reinforced epoxy composites dampen better compared to glass and carbon fibre composites. The damping of hemp fibre- and flax fibre-polypropylene (PP) composites is greater compared to glass fibre composites, as stated in [4]. The damping rises with flax fabric crimps and flax yarn twist angles, related to increased inter-yarn and intra-yarn frictions, respectively [2,5], but higher fabric crimps decrease the tensile modulus [14]. The damping of carbon fibre- and flax fibre-epoxy composites also rises as the angle of the ply orientation increases, with flax fibre composites showing a 2–3 times greater loss factor than carbon fibre composites [6]. Idicula et al. [7] investigated the impact of fibre content on damping in composites made of banana fibres, sisal fibres, and PS (polyester). They showed that the maximum damping could be observed for composites containing a fibre content of 40% by volume. Two studies [8,10] suggested that the inclusion of sisal fibres lowers the composite damping more than PP and PS (polystyrene) specimens, respectively. Measurements with low frequencies show reduced damping in the case of banana fibre-PS composites compared to high frequencies [11]. In another investigation, Guen et al. [9] indicated that flax fibre-epoxy composites have less damping in comparison to polyol-treated flax fibre composites. The composite samples with poor interface bonding between fibres and the matrix tend to dissipate more energy than the samples with good interface bonding, as pointed out in [12]. Moreover, the frequency has a significant effect on the energy dissipation of the composite [13].

The DMA experiments were typically carried out at different temperatures with a fixed frequency equal to or less than 100 Hz in the references (i.e., [2,3,4,5,6,7,8,9,10,11,12,13]) mentioned above. With excitation frequencies of up to 200 Hz, Etaati et al. [15] measured damping on hemp fibre-PP composites. The maximum damping is reported for composites with a hemp fibre weight fraction of 0.30, and the loss factor has a little frequency dependency at less than 30 Hz.

Plant fibre composites can be considered as potentially useful materials for interior applications in transport [16] to reduce noise and vibration. In vehicles, for example, low-frequency sound in the range of 10–160 Hz is important and can cause noise annoyance and adverse mental performance [17]. Thus, the ability to add damping over the low-frequency range is necessary, and this provides the motivation in this study to measure damping over a low-frequency range of up to 100 Hz. The dynamic behavior, such as the damping of flax fibre-reinforced polypropylene composites (FFPCs), has not previously been reported over the frequency range of 100 Hz using DMA. The present work is, therefore, an attempt to measure the dynamic characteristics of FFPCs up to frequencies of 100 Hz.

The alignment of fibres (i.e., fibre orientation) in composites is important to make them suitable as structural materials. Plant fibres are discontinuous and readily available from the textile industry in the forms of yarns/rovings and aligned fabrics, which implies a continuous product with highly controlled fibre orientation. Therefore, by applying aligned or UD (unidirectional) plant fibre fabrics as reinforcement for a composite, the full potential of plant fibres (e.g., flax) can be realised and form the essential foundation from which the potential of plant fibres in structural applications can be identified. The properties of composites typically vary depending on the fibre content, fibre type, fibre aspect ratio, fiber orientation, fibre surface treatment, polymer matrix type, and processing conditions. However, it is expected that the stiffness, strength, and deflection resistance of composites will increase with adding fibres. In addition, plant fibre composites can have improved biodegradability and lower environmental impact compared to polymers.

In general, estimating the tensile properties of a uniaxial composite is adequate to know the reinforcing contribution of plant fibres. However, the structure may experience not only uniaxial loads but also off-axis loads. Because of the composites’ anisotropicity, off-axis loads have a significantly adverse impact on their mechanical properties. However, the mechanical characteristics of UD flax/PP composites under off-axis loads have been infrequently studied. If FFPCs are to be seriously considered for structural applications, their responses to off-axis loads need to be thoroughly investigated and documented in an array of different loading conditions (e.g., tension and bending).

The previously reported tensile and flexural properties of aligned plant fibre composites are mainly based on experiments in the axial direction (i.e., the fibre direction). However, several studies have analysed various off-axis loadings for plant fibre composites. For example, the off-axis (15°, 30°, 45°, 60°, and 75°) and axial (i.e., 0°) tensile stiffness of UD sisal/epoxy composites was investigated by Ntenga et al. [18]. Madsen et al. [19] examined the mechanical properties (tensile modulus, tensile strength, and tensile strain at break) of UD hemp/PET composites in various directions (0°, 10°, 20°, 30°, 45°, 60°, and 90°). They found that the tensile characteristics (stiffness and strength) drop considerably when the loading angle increases.

There are several investigations where flexural and tensile characteristics (stiffness and strength in both the transverse and longitudinal directions) of UD flax/epoxy [20,21,22,23], tensile behavior (stiffness and strength in both the transverse and longitudinal directions) of flax yarn/PP [24], UD flax/PLA (poly-(lactic acid)) [25], UD flax/biopolymers (poly-(hydroxy alkanoate), poly-(lactic acid), and (poly-(butylene-succinate)) [26], and tensile performance (stiffness and strain in the transverse direction) of UD flax/polyester [27] composites are measured. However, there is no work published in accessible journals or dissertations that estimates the tensile and flexural characteristics of continuously aligned flax reinforced-polypropylene composites for a variety of loading angles. Therefore, all laminates are evaluated in the axial (0°) and transverse (90°) directions in this work, and three other laminates (30°, 45°, and 60°) are produced to estimate the intermediate off-axis performances in terms of tensile and flexural properties. Failure modes of the fracture samples are also analysed using an environmental scanning electron microscope (ESEM). Additionally, the comparison of damping, tensile, and flexural properties of FFPCs is made with glass fibre composites. This is to evaluate the potential of flax fibres as a natural substitute to synthetic glass fibres for various structural and non-structural applications.

## 2. Experimental Details

### 2.1. Materials

Sheets of PP random copolymer (MOPLEN RP241G) with a melt flow rate of 1.5 g/10 min, determined by ISO 1133, and a thickness of 0.38 mm were used as matrix material. The polypropylene sheets were produced by Lyondell Basell Industries and supplied by Field International Ltd., Auckland, New Zealand. The properties of PP are shown in reference [28]. Unidirectional flax fabric (FlaxPly UD180) with a nominal specific weight of 180 g/m^2^ and density of 1.42 g/cm^3^ was used as reinforcement. The flax fabric (42.5 yarns/cm (warp) and 3 yarns/cm (weft)) was supplied by Lineo, Meulebeke, Belgium. The weight distribution of flax fabric in the warp and weft directions was 95.5% and 4.5%, respectively, according to the manufacturer’s datasheet. The role of weft (or fill) yarns is to keep the warp yarns together.

### 2.2. Manufacturing of Composites

**Figure 1:**
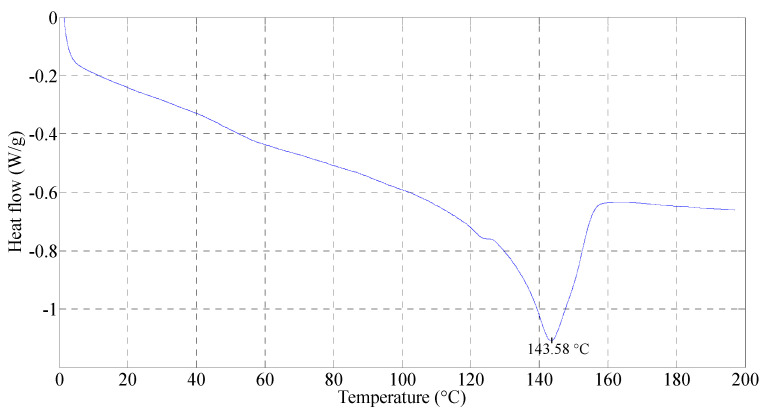
A differential scanning calorimetry curve of neat PP.

A vacuum bagging technique was used to manufacture the composite samples. The consolidation temperature was determined based on the differential scanning calorimetry curve of PP, as shown in Figure 1, which shows the peak melting temperature of 143.58 °C. One can estimate that the processing temperature has to be at least 40 °C above the melting temperature of the polymer in order to have the lowest viscosity [29,30,31]. However, the degradation of flax fibres starts above 200 °C with the degradation of the hemicellulose [32,33]. Therefore, a consolidation temperature of 190 °C was chosen to avoid the degradation of the flax fibres.

Flax fabrics were dried for 24 h at 70 °C in a vacuum dryer (Squaroid duo-vac vacuum oven) to reduce the moisture content. Dry flax fabrics and PP sheets were interleaved by a hand lay-up process and placed on an aluminum plate. Then, a peel ply was used to separate the breather from the laminate, and the breather was employed to ensure all the air inside the vacuum bag could be drawn into a vacuum port. After sealing the material stack, the air was evacuated from inside the vacuum bag using a vacuum pump. The mold was subsequently placed inside the Elecfurn (FAC 100) oven and heated to 190 °C for 1 h. After this, the mold was cooled to a room temperature of around 24 °C. The temperature-pressure cycle (cycle 1) was applied according to the reference [34]. The panels’ nominal size was 600 mm × 500 mm, with a target thickness of 3 mm and target fibre volume fractions of 0.31, 0.40, and 0.50. The number of layers of PP sheet and flax fabric used for different fibre volume fractions is shown in Table 1. Neat PP samples were also manufactured for comparison with the respective flax/PP samples. It is to be noted that no additives were added for better adhesion between the matrix and reinforcement. This is because fibre surface treatment with chemical reagents can be expensive (e.g., silanes) and/or toxic (e.g., isocyanates), tarnishing the low-cost, eco-friendly image of plant fibres [35], and improvements in interfacial properties often lead to a reduction in toughness [30] and vibration damping [36].

#### Orientation in Unidirectional FFPCs

**Figure 2:**
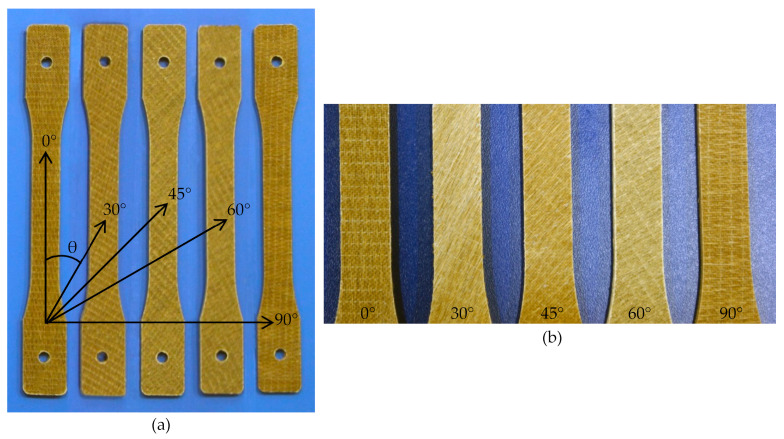
(**a**) Tensile samples with the fibre axis inclined at different angles to the testing direction and (**b**) fibre orientation (clear view).

Figure 2 shows the tensile samples loaded with the fibre axis inclined at various angles (0°, 30°, 45°, 60°, and 90°) to the testing direction. The fibre orientations correspond to the defined angles in the figure (Figure 2a), and the orientation of the fibre axis can be clearly identified from the texture of sample surfaces (Figure 2b). Importantly, these off-axis orientations play a major role in determining the mechanical properties of composites.

### 2.3. Dynamic Mechanical Analysis 

Dynamic mechanical analysis (DMA) was performed using a dynamic mechanical analyser (TA Instruments, model: Q800, New Castle, USA). The tests were conducted in the single cantilever mode to characterise the response of composite samples under flexural vibration. The specimens were cut from the composite panels with a size of 35 mm × 12.70 mm × thickness of the material using an automatic saw. A single-frequency sweep test was made over a frequency range of 0.01 to 200 Hz. The experiment held the sample at about 21 °C and performed a frequency sweep at a constant strain of 0.05%. Five specimens for each test were used to ensure consistency in results. The storage modulus and loss factor were then obtained. Although the measurements were carried out up to frequencies of 200 Hz, the measurements between 100 Hz and 200 Hz are not shown due to their inaccuracies. This is believed to be due to the resonance or artefact of the DMA instrument. The frequency range considered for analysis was 5 to 100 Hz.

### 2.4. Tensile Test

The tensile properties of FFPCs were determined using an Instron universal testing machine (Instron 5567, Norwood, MA, USA) with a 30 kN load cell according to ASTM D638-10. A mechanical extensometer (model 2630-112) was used with a nominal gauge length of 50 mm to measure the strain. A crosshead speed of 5 mm/min was applied. Samples were cut by a five axes CNC (computer numerical control) milling machine (model DMU 50) to prepare standard dog-bone-shaped test specimens (Type I) following the dimensions specified in the standard. The modulus measurement was performed by the Instron Bluehill material testing software using the slope of a chord in the strain range of 0.05–0.25%, as specified in ASTM D638. The properties of at least five specimens were averaged for each sample as per the standard. All tests (i.e., tensile and flexural) in this study were conducted at an ambient temperature of about 21 °C and a relative humidity of 48%. This relative humidity does not affect the mechanical characteristics or composite structure since the critical relative humidity ranges between 75% and 98%, above which increases in moisture content irreversibly reduce the mechanical properties of the composite [37].

### 2.5. Flexural Test

Flexural properties were measured by a three-point bend test using an Instron 5567 with a 10 kN load cell following ASTM D760-10. The support span length and crosshead speed (procedure A in the standard) were calculated based on the specimen thickness. The specimens were cut from the composite panels with a dimension of 75 mm × 12.7 mm × thickness using an automatic saw. The flexural strength was estimated at 5% strain, as per the standard. The average value and standard error obtained from at least five test specimens were reported.

### 2.6. Morphological Characterisation

Morphologies of fracture cross-sections after tensile tests were investigated using the ESEM (environmental scanning electron microscope) (FEI Quanta 200F, Hillsboro, OR, USA). Cross-sections were used to get an accurate representation of the internal microstructure of specimens. The pieces of specimens were mounted on aluminum stubs or brass bits. All specimens were then coated for approximately 20 min with a very thin layer of fine platinum by a compact rotary-pumped sputter coater (Q150R S, Quorum Technologies, Laughton, UK) in order to aid electron conductance. After this, the ESEM micrographs of the specimens were captured.

### 2.7. Measures of Void Fraction

Optical microscopic images of the cross-section (~3 mm × 6 mm) of flax/PP samples were captured using micro-computed tomography (micro-CT). These images were then analysed using the image analysis software ImageJ [38] to determine the percentage of voids. In this analysis, the image was converted to a binary image, and thus the fibre and matrix and the voids were converted to white and black pixels, respectively. After this, the image was analysed using built-in software tools, which estimated the total number of voids and their overall area fractions. The mean void fraction and standard deviation obtained from three specimens of each fibre volume fraction were reported.

## 3. Results and Discussion

### 3.1. Dynamic Characteristics

#### 3.1.1. Storage Modulus

##### Effect of Fibre Content

**Figure 3:**
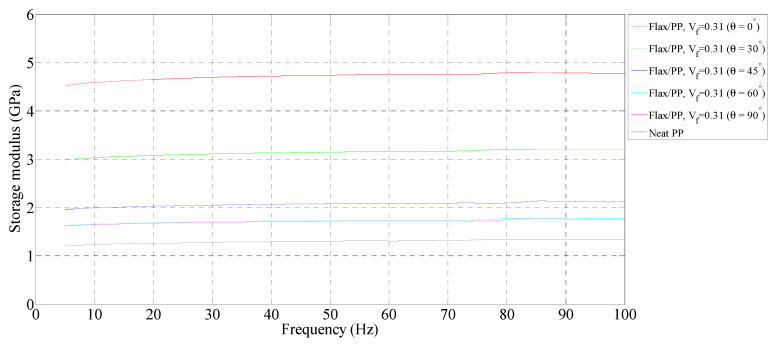
Storage modulus of flax/PP composite samples of different fibre orientations for a fibre volume fraction of 0.31 and a neat PP sample.

**Figure 4:**
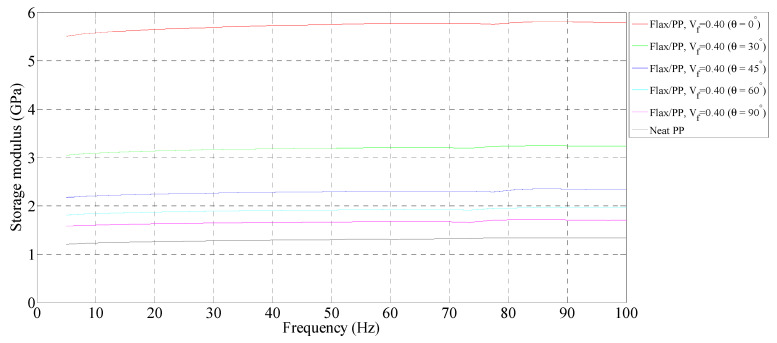
Storage modulus of flax/PP composite samples of different fibre orientations for a fibre volume fraction of 0.40 and a neat PP sample.

**Figure 5:**
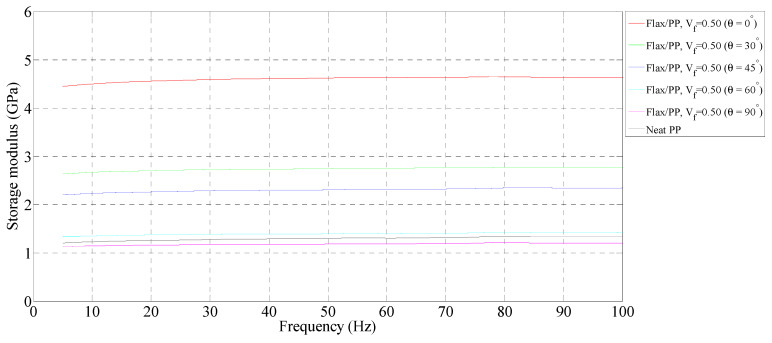
Storage modulus of flax/PP composite samples of different fibre orientations for a fibre volume fraction of 0.50 and a neat PP sample.

**Figure 6:**
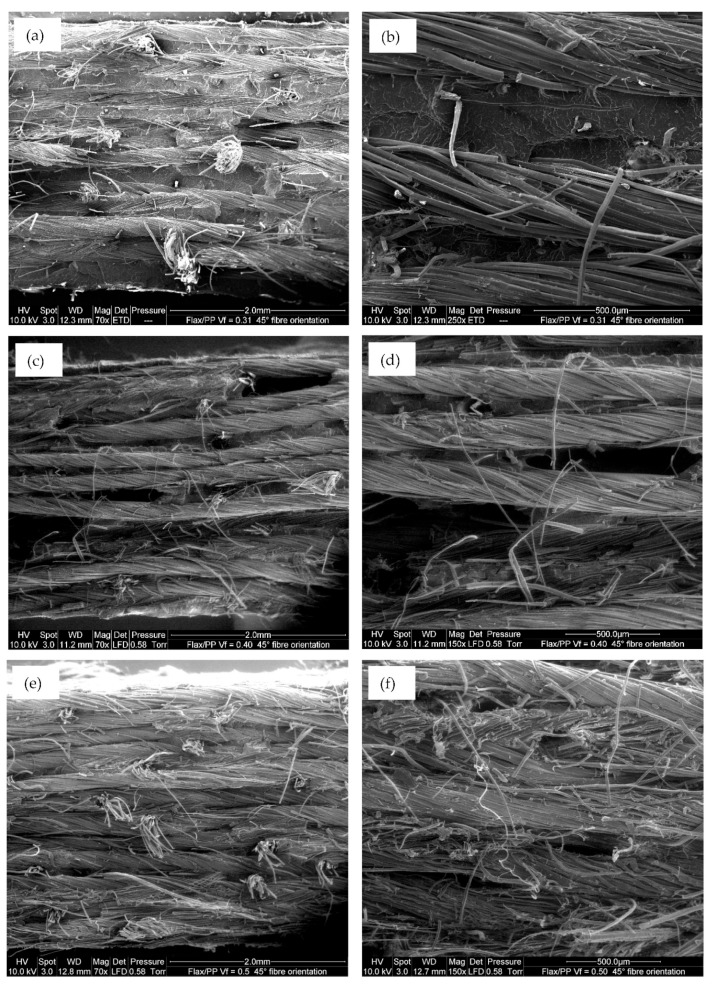
ESEM microimages of fracture samples: (**a**,**b**) showing no fibre-fibre contact (*V_f_* = 0.31 and *θ* = 45°), (**c**,**d**) showing insignificant fibre-fibre contact (*V_f_* = 0.40 and *θ* = 45°), and (**e**,**f**) showing significant fibre-fibre contact (*V_f_* = 0.50 and *θ* = 45°), respectively, at two different magnifications.

Figure 3, Figure 4 and Figure 5 show the storage moduli of composite and neat PP samples. There is a substantial increase in the modulus of PP with the incorporation of flax fibres, particularly in the 0° fibre orientation, over the considered frequency range. This is due to the increase in matrix stiffness with the reinforcing effect imparted by the fibres allowing a greater degree of stress transfer at the interface [8,39,40]. These results are consistent with those obtained by other authors [15,41]. For a given fibre orientation, the storage moduli increase up to a fibre loading of 40% by volume, and then it is observed to decrease with a further increase in the fibre content. A similar result was also reported by Idicula et al. [7] in the case of banana/sisal hybrid fibre-reinforced polyester composites. This is because of an increase in fibre-to-fibre interaction for composites with a 50% fibre loading by volume (Figure 6e,f) relative to composites with 31% (Figure 6a,b) and 40% (Figure 6c,d) fibre loading by volume. This results in diminished effective stress transfer between the matrix and fibre.

##### Effect of Fibre Orientation

The storage moduli decrease with increasing fibre orientation at each fibre volume fraction, with a maximum reduction between 0° and 30° fibre-oriented samples. This is due to the high stiffness of composites in the fibre direction (i.e., 0°). Hadi and Ashton [42] reported similar observations in the case of glass/epoxy composites. The 0° fibre-oriented samples have the maximum storage modulus, while the 90° samples have the minimum modulus in all fibre volume fractions. The maximum storage modulus of 5.73 GPa, on average, is found for a 0° fibre orientation (*V_f_* = 0.40), whereas the minimum modulus of 1.18 GPa, on average, is observed for a 90° fibre orientation (*V_f_* = 0.50).

##### Effect of Frequency

There is a slight increase in the storage moduli of composite and neat PP samples with frequency. In particular, the rise in storage moduli is in the range of 4–10% for the composite samples, while the neat PP shows an increase of 11%. The maximum increase in storage modulus (10%, from 1.62 GPa to 1.78 GPa on average) is seen in the 60° fibre-oriented sample (*V_f_* = 0.31). At each fibre orientation, the storage modulus tends to increase because of the lower mobility of polymeric chains at a higher frequency [43]. The modulus measurements carried out over shorter times (high frequency) lead to higher values, whereas measurements taken over longer times (low frequency) lead to lower values [44]. This is also owing to the rearrangement of material at the molecular level and the displacement of molecules relative to each other under dynamic stresses. Joesph et al. [8] and Pothan et al. [11] claimed that if a material is subjected to constant stress, its modulus changes over time due to the material undergoing rearrangement in an attempt to minimise the localised stresses.

Overall, adding more flax increases the storage moduli up to a fibre volume fraction of 0.40, with a maximum storage modulus of 5.73 GPa. An increase in storage modulus indicates higher stiffness. The storage moduli decrease with increasing fibre orientation for a given fibre volume fraction. As the frequency increases, the storage modulus increases in the range of 4–10%.

#### 3.1.2. Loss Factor

##### Effect of Fibre Content

**Figure 7:**
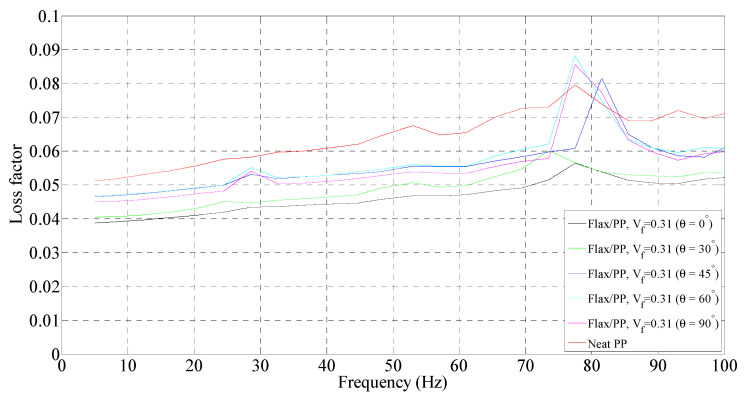
Loss factor of flax/PP composite samples of different fibre orientations for a fibre volume fraction of 0.31 and a neat PP sample.

**Figure 8:**
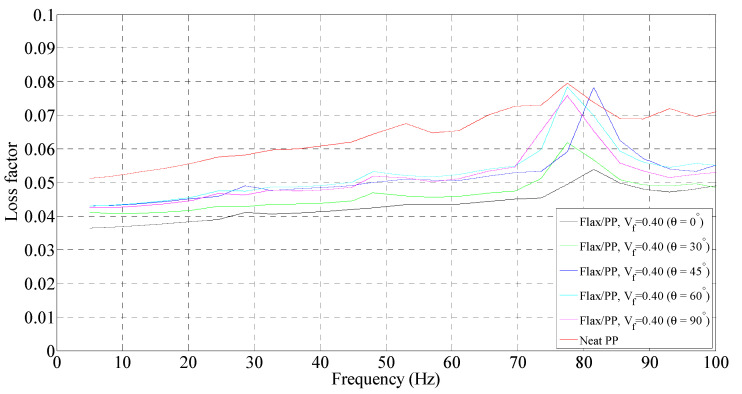
Loss factor of flax/PP composite samples of different fibre orientations for a fibre volume fraction of 0.40 and a neat PP sample.

**Figure 9:**
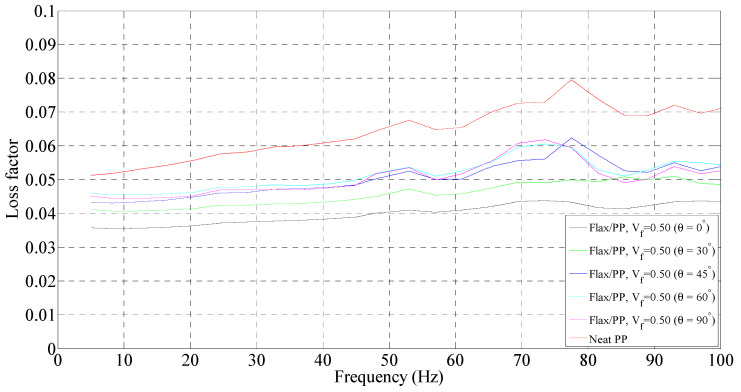
Loss factor of flax/PP composite samples of different fibre orientations for a fibre volume fraction of 0.50 and a neat PP sample.

**Figure 10:**
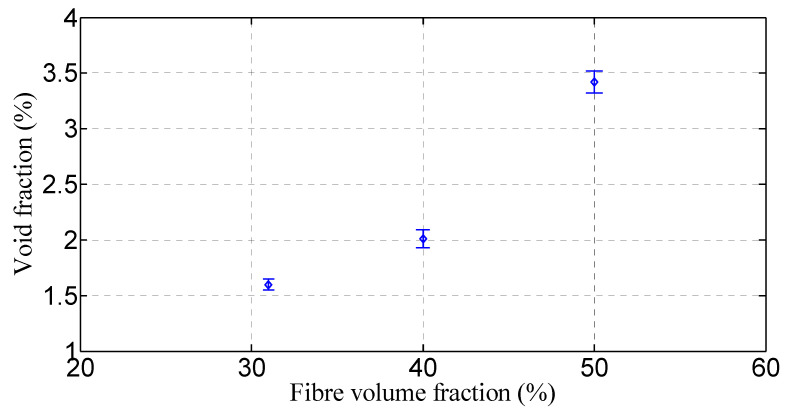
Void content of flax/PP composites.

**Figure 11:**
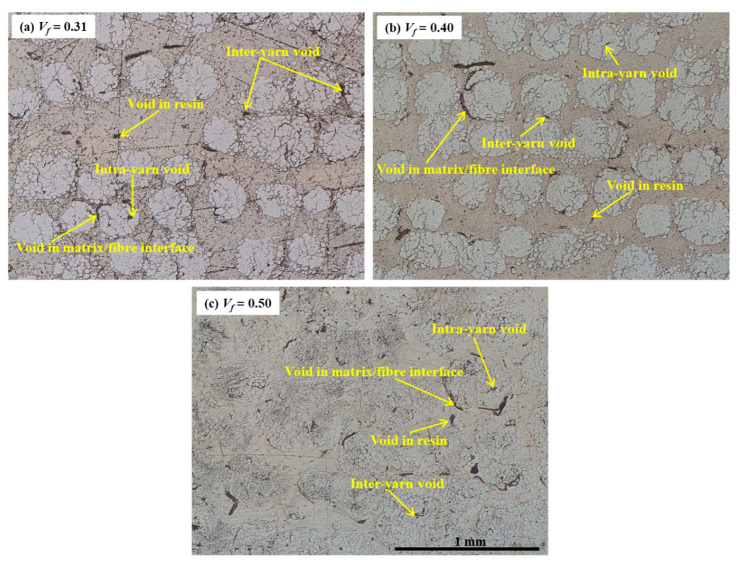
Micro-CT cross-section images of flax/PP composites showing the voids in the matrix and matrix/fibre interface, and the intra-yarn and inter-yarn voids.

The variation in loss factors for the composite and neat PP samples is shown in Figure 7, Figure 8 and Figure 9. In general, the incorporation of flax fibres decreases the loss factor in comparison with the neat PP matrix. However, there is no noticeable difference in the loss factors with increasing flax fibre. In particular, the composite samples with 40% and 50% fibre contents by volume show almost the same loss factors as those of 31% by volume. The loss factors lie mostly in the range of 4.0–5.5% for fibre orientations of 30°, 45°, 60°, and 90°, and 3.5–4.5% for fibre orientation of 0° up to frequencies of 60 Hz. This is probably owing to the increased overall interface regions inside composites as the fibre content increases. The greater the interface regions, the greater the energy dissipation sites, resulting in enhanced energy dissipation. An increased energy dissipation with growing interfacial areas balances out some of the potential loss due to a reduced amount of viscoelastic polymer. The presence of voids in the composite also contributes to the high damping. The void content is found to be in the range of 1.60–3.42% for the composites with different fibre volume fractions, as shown in Figure 10. The void content increases with an increase in the fibre volume fraction, owing to a rise in the fibre surface area. Voids are seen in the matrix and matrix/fibre interface, within the yarn (intra-yarn void), and between adjacent yarns (inter-yarn void) (see Figure 11). They are caused by the luminal cavities and other hollow features within fibres/yarns, compressibility, low wettability of plant fibres, and the presence of air during processing [45]. Two other studies [46,47] also mentioned that air entrapment or voids in fibres are often caused by inadequate wetting or impregnation with resin. Moreover, energy dissipation in composites occurs due to the internal friction and hysteretic behavior of the constituents (i.e., fibres and matrix). When a composite is subjected to cyclic loading, energy is absorbed and converted into heat, reducing the amplitude of vibrations [30,36,48]. Apart from the fabrication-induced defects [49], the inherent heterogeneity and non-uniform distribution of plant fibres result in a complex network of microcracks and damage accumulation, thereby increasing internal friction and energy dissipation [36]. Damage may also initiate in composites and propagate within the fibres themselves due to the hierarchical structure of flax fibers, which contain stacks of layers reinforced by microfibrils [50], eventually causing energy dissipation. Furthermore, the viscoelastic nature of fibres contributes to energy dissipation. These are the plausible factors for generating nearly the same damping, despite a reduction of the polymeric matrix.

##### Effect of Fibre Orientation

For a given fibre volume fraction, the 30°, 45°, 60°, and 90° fibre-oriented samples show higher loss factors compared to the 0° sample. The low loss factor for the 0° sample could be because of the higher storage modulus compared to other fibre-oriented samples (see Figure 3, Figure 4 and Figure 5), while the loss modulus is not relatively higher. However, the sample with fibre orientations of 45° and 60° show the highest loss factors. This is because the total energy is dominated by the in-plane shear strain energy [51], which is maximised at these fibre orientations in FFPCs. The loss factors lie in the range of 4.3 to 5.5%, irrespective of the fibre volume fraction. Interestingly, the apparent peaks of the samples for different fibre volume fractions and orientations appear between 70 to 90 Hz. Once again, this is believed to be due to the resonance or artefact of the DMA instrument.

##### Effect of Frequency

The loss factor is found to increase with frequency for a given fibre orientation. The loss factor of neat PP exhibits an increase of 28% (from 0.051 to 0.065) up to frequencies of 60 Hz (see Figure 7). A maximum rise in loss factor of 28% (0.043 to 0.055) is observed for a fibre volume fraction of 0.50 and orientation of 45°. The increase is in the range of 14–28% over the frequency range of 5 to 60 Hz for all fibre volume fractions and orientations. The increase in damping with frequency may come from fibre/fibre and fibre/matrix interactions [52]. Molecular motions at interfaces also contribute to the damping of the composite in addition to those of constituents [53,54].

Overall, the loss factors predominantly lie in the range of 4–5.5%, 4–5.3%, and 4–5.2% for fibre volume fractions of 0.31, 0.40, and 0.50, respectively, over the frequency range of 5 to 60 Hz. Samples with fibre orientations of 45° and 60° show higher damping compared to other fibre orientations (0°, 30°, and 90°). As the frequency increases, the increase in the loss factor is 14–28%. However, there is no significant difference in loss factors among the composites with different fibre contents. The loss factors lie mainly in the range of 4–5.5%, irrespective of the fibre content and orientation, up to frequencies of 60 Hz.

##### A Comparison with Glass Fibre Composites

The damping ranges from 0.38 to 1.39% for different fibre orientations (from 0° to 90° with 15° increments) for glass fibre/epoxy composites (*V_f_* = 0.40) up to frequencies of 60 Hz, as reported by Mahi et al. [55]. Whereas, in this investigation, the damping is seen to be in the range of 4 to 5.3% for flax/PP composites (*V_f_* = 0.40), demonstrating that the damping of flax fibre composites is significantly higher (281 to 953%) than glass fibre composites. This is because of the intrinsic porosity (0.4 to 7.2% [56] and 0 to 7.2% [57]), viscoelastic nature of plant fibres, and differences in the matrices. Notably, in general, the loss factors of PP and epoxy are 0.06 [30,58,59,60,61] and 0.02 [2], respectively. For the former (i.e., glass fibre/epoxy), the increase in damping is 2–5% with increasing frequency (0 to 60 Hz), regardless of the fibre orientation, whereas, for the latter (i.e., flax fibre/PP), the increase is 14–28%. Thus, much better damping can be obtained in flax/PP composites compared to most widespread glass/epoxy composites.

### 3.2. Mechanical Characteristics

#### 3.2.1. Tensile Properties

**Figure 12:**
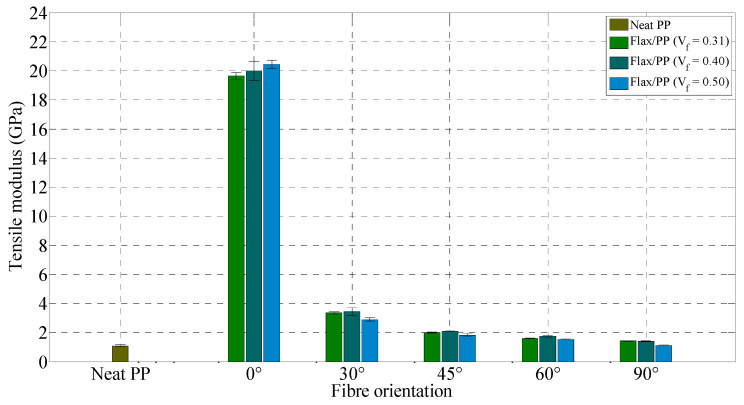
Tensile modulus for neat PP and flax/PP samples of different fibre volume fractions and orientations.

**Figure 13:**
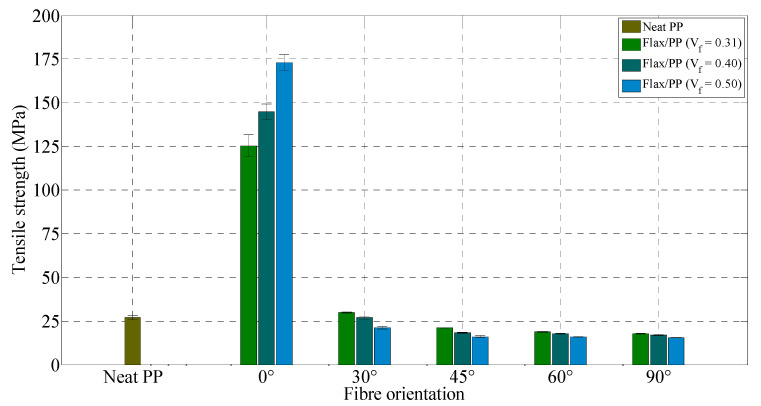
Tensile strength for neat PP and flax/PP samples of different fibre volume fractions and orientations.

Tensile moduli and strengths of flax/PP composites, along with neat PP, are shown in Figure 12 and Figure 13, respectively. The tensile modulus of composites increases with fibre content for all fibre orientations up to a fibre volume fraction of 0.40, and then it is seen to decrease with the inclusion of more fibres, except for the 0° fibre orientation. As the fibre volume fraction increases, the tensile strength for a 0° fibre orientation increases, while for the other fibre orientations, it drops slightly. This variation in trend is perhaps due to the higher dependency of stiffness on composite strength rather than interfacial strength. This is also because all the fibres can contribute to the composite’s stiffness and carry the load in the case of samples with a 0° fibre orientation. Moreover, with increasing fibre content, the strength can reduce (modulus may still increase) due to the presence of more fibre/matrix interfacial areas where incompatibility of hydrophobic PP and hydrophilic fibres plays an important role. However, the degree of fibre/matrix compatibility can be improved using coupling agents [62,63,64], chemical/physical treatments [65,66], nanomaterial treatments [67,68,69], or polymer grafting [70].

It is observed from Figure 12 and Figure 13 that the tensile modulus and strength of composites drop significantly between 0° and 30°. However, there is little variation in composite modulus and strength between 45° and 90°. This trend is similar to that stated by other authors [71,72]. The tensile properties of the specimens are highly influenced by the fibre orientation (with respect to the loading direction); for instance, tensile modulus and strength are reduced from 16.66 GPa to 3.36 GPa and 125 MPa to 29 MPa, respectively, at an off-axis angle of 30° (*V_f_* = 0.31). The decrease in composite modulus and strength by increasing fibre orientation is due to the decline in the fibres’ contribution to carrying the applied load. Respectively, the tensile modulus and strength of composites loaded in the 0° fibre orientation (*V_f_* = 0.40) are 13.70 and 8.50 times higher than the tensile modulus and strength of 90° fibre orientation (*V_f_* = 0.40). The minimum tensile properties are found for a 90° fibre orientation, where the properties are dominated by the matrix material rather than the fibres, as the fibres lie perpendicular to the direction of the applied load.

The maximum tensile modulus (20.44 GPa) and strength (173.08 MPa) of the samples are, however, observed for a fibre volume fraction and orientation of 0.50 and 0°, respectively, while the neat PP shows a tensile modulus and strength of 1.11 GPa and 27.06 MPa, respectively. Such a significant improvement in composite tensile properties occurs due to the effective stress transfer between fibres and PP at this particular fibre orientation. The specimens with a 0° fibre orientation also offer a minimum chance of shear failure (discussed in Section 3.2.2) and thus provide a better load-carrying capability than other fibre orientations. The tensile modulus and strength of neat PP are found to be in the range of 0.95–1.77 GPa and 26–41.4 MPa, respectively, as quoted in the literature [73].

Generally, a composite shows a maximum stiffness up to fibre contents of 55–65 weight% [45,74] and strength peaks with fibre contents of 40–50 weight% [75] if sufficient interfacial bonding strength between fibres and matrix is ensured (in this study, 31% by volume ≈ 40% by weight, 40% by volume ≈ 50% by weight, and 50% by volume ≈ 60% by weight). The specimens with a fibre orientation of 0° (*V_f_* = 0.50) show the highest tensile modulus and strength. However, similar behaviour in terms of modulus and strength is not observed for the rest of fibre orientations. This can be because no conventional coupling agents or any chemical modifications are used. Hence, the interfacial strength between the fibre and the matrix is poor, which results in low tensile properties of the resulting polymer-based composites.

Plant fibres are used in this study as yarns, where the individual fibres are bonded together with lignin and/or pectin, and yarns have lower mechanical properties than individual fibres due to low bonding strength between the individual fibres [76]. Another negative impact of yarns is that fibre tightens within the yarns due to twisting, thus making resin impregnation difficult [77]. Fibres are twisted to some degree, as shown in Figure 6. Therefore, fibres are not perfectly aligned with the direction of the applied load and thus contribute negatively to the mechanical properties of the resultant composites. Gill [78] mentioned that even an insignificant percentage of twisting within the fibres is detrimental because the twisted fibres cannot fully contribute to the strength but provide an irregular matrix distribution. A twist angle of 20° can decrease the stiffness by 50% in flax fibre composites (*θ* = 0°) [79], as fibres are not aligned with the loading direction. However, because plant fibres are discontinuous, twisting is required to achieve strength (or weaving and use as a textile) [50].

**Figure 14:**
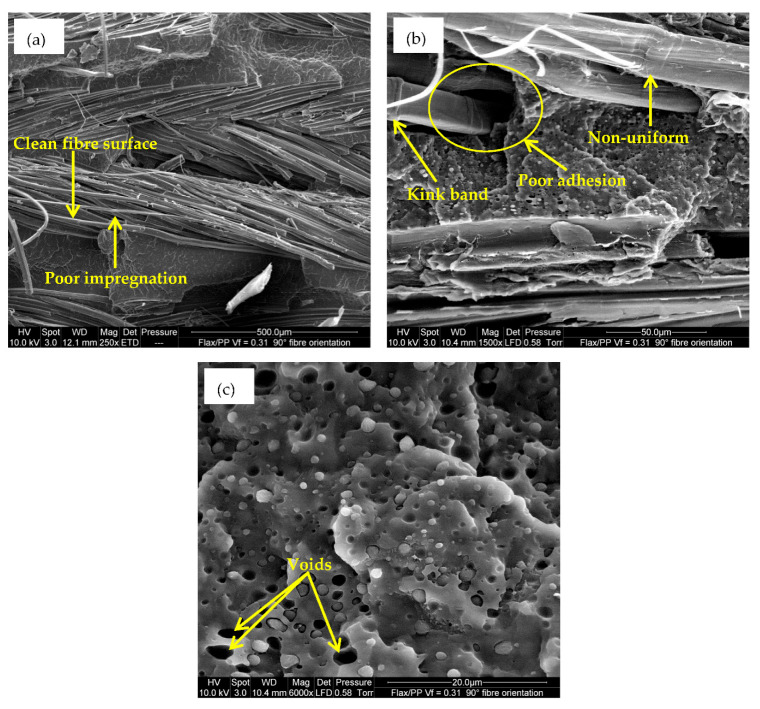
(**a**) Poor impregnation of yarn by the matrix, (**b**) poor adhesion between flax fibres and PP, and (**c**) voids in a matrix-rich region.

The homogeneous distribution and dispersion of fibers in the polymer matrix greatly influence the mechanical properties. In Figure 14a, there are many fibres that are not fully bonded to the matrix material, adversely affecting the mechanical properties of the composites. Furthermore, voids (see Figure 11 and Figure 14c) can also contribute towards lower tensile properties as they increase with increasing fibre content (see Figure 10). An earlier study found that void content can reach 4% by volume for a fibre content of 60% by weight [24]. The rough surface and the twisted structure of the flax yarns are responsible for creating the voids. Their location can be between the fibre/matrix interfaces, between the flax yarns, or inside the yarns [80].

**Figure 15:**
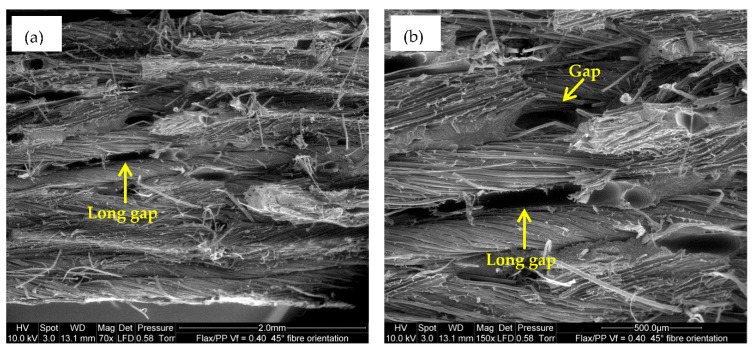
ESEM images of tensile fracture samples: (**a**,**b**) showing a long gap for a fibre volume fraction and orientation of 0.40 and 45°, respectively, at two different magnifications.

For a high fibre volume fraction (*V_f_* = 0.50), as more fibre-to-fibre interaction occurs (see Figure 6e,f), the result is a reduction in tensile properties. In addition, the surfaces of the fibres are clean (Figure 14a), and in some instances, there are small gaps between the fibres and matrix (Figure 14b), which is evidence of insufficient fibre wetting. This is perhaps because PP is not sufficient to entirely wet the 12 layers of flax fabrics in the composite (*V_f_* = 0.50). This results in poor fibre/matrix adhesion, leading to a reduction in tensile properties at high fibre loading. Moreover, inadequate adhesion between flax fibres and matrix stimulates small crack formation at the interface and non-uniform stress transfer due to fibre accumulation within the matrix [81]. The ESEM images in Figure 15a,b also reveal a long gap, suggesting poor bonding between flax fibres and PP, and it has some deleterious impact on the mechanical properties.

#### 3.2.2. Failure Strains and Failure Modes

**Figure 16:**
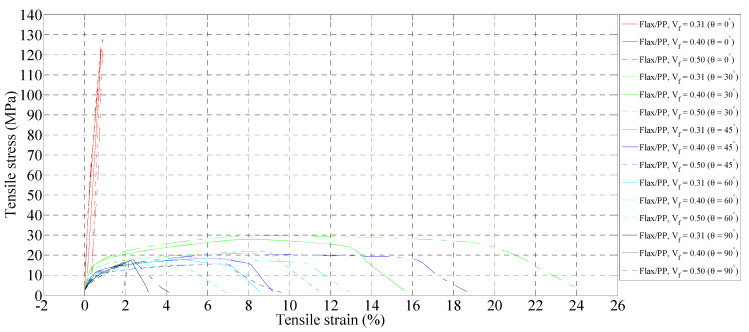
Representative stress-strain curves of flax/PP composite samples with different fibre volume fractions and orientations.

Figure 16 shows stress-strain curves for flax/PP composite samples of various fibre volume fractions and orientations. These curves display the overall changes in tensile modulus and strength of the samples with increasing fibre volume fraction and orientation. The curves are shifted downward as fibre orientation increases, indicating that the tensile properties decrease.

The stress-strain curves can be roughly defined into three zones. All specimens within the initial region, up to 0.25% strain, show almost a linear relationship between stress and strain, during which modulus estimation is performed. After that, the curves in the second region demonstrate non-linear behaviour before reaching the maximum tensile stress. When the maximum stress is reached, the curves present a decreasing trend considered as the third region. In this regard, the trends of the curves do not seem to be similar for all fibre orientations. For 0° fibre orientation, in particular, the post-peak curves go down very quickly while not increasing in strains. This means that the specimens break into two pieces once the maximum stress is reached. In contrast, for composites with fibre orientations of 30°, 45°, 60°, and 90°, the post-peak curves drop with a continuous increase in strains; this exhibits a ductile behaviour before breakage of the specimens. This is perhaps because the specimens are broken into pieces after reaching the maximum stresses, while some fibres are not broken into two pieces, withstanding the applied stresses to some degree.

**Figure 17:**
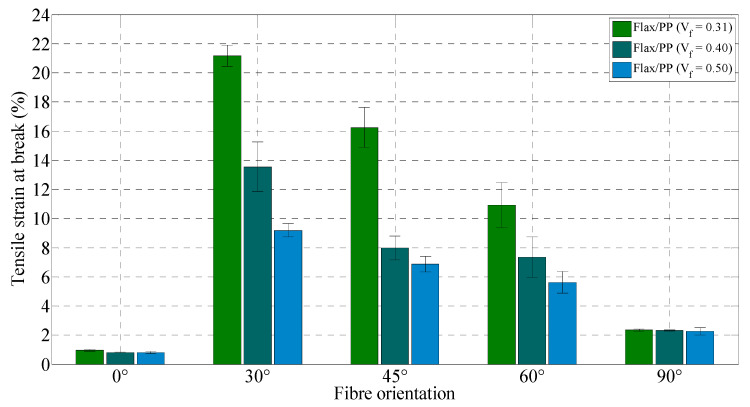
Tensile strain at break for flax/PP samples of different fibre volume fractions and orientations.

The tensile strains at break of the composites are summarised in Figure 17. The tensile strains at failure decrease with increasing fibre volume fraction. Other authors also reported the same trend in their studies [46,82,83]. This is perhaps because of the embrittlement effect, as it increases with increasing fibre content. In addition, an increase in fibre content leads to more fibre/matrix interfaces. Therefore, crack propagation becomes easier, and the failure strain becomes smaller for high-fibre content [27]. The specimens with a 30° fibre orientation demonstrate higher tensile strain compared to other fibre orientations (0°, 45°, 60°, and 90°), and the maximum strain at failure of 21.17% is observed for a fibre volume fraction and orientation of 0.31 and 30°, respectively. In another study, Mortazavian and Fatemi [84] observed that tensile strain at break is higher for 45° than 0° and 90° samples in the case of glass fibre/polybutylene terephthalate composites. The authors did not consider any other fibre orientations. This is because of the strain limit of fibres, and fibres are aligned with the direction of loading in the case of a 0° oriented sample; hence, the low capability of fibres in straining creates a low ductility of the composite [84]. With the increase in fibre orientation relative to the test direction (i.e., *θ* = 0°), the failure is more influenced by the matrix and interfacial properties. Therefore, for composites with fibre orientations of 30°, 45°, 60°, and 90°, matrix straining is more influential compared to 0°. The deformed matrix has more room to elongate between fibres and in the loading direction [84] for 30° specimens in comparison to 45°, 60°, and 90° specimens. Thus, the 30° specimen’s high tendency to show ductile behaviour leads to a higher failure strain.

For 30° fibre orientation, the tensile strain at break decreases by 130% (21.17% to 9.2%) when the fibre volume fraction increases from 0.31 to 0.50. Composites loaded in the on-axis orientation (0°) have a minimum failure strain, while composites loaded in the off-axis orientation (30°) have a maximum failure strain irrespective of the fibre volume fraction and orientation. The failure strain drops significantly at fibre loadings of 31% and 40% by volume between 30° and 90°. The decrease is slight at a fibre loading of 50% by volume. Notably, the standard deviations of tensile modulus and strength of five replicated specimens of flax composite range from 0.02 to 0.31 GPa (Figure 12) and 0.44 to 4.84 MPa (Figure 13), respectively, regardless of fibre contents and orientations, while they are in the range of 0.02 to 1.71% for tensile strain at break (Figure 17). Most measurements have minimal standard deviations, suggesting consistency in measurements.

**Figure 18:**
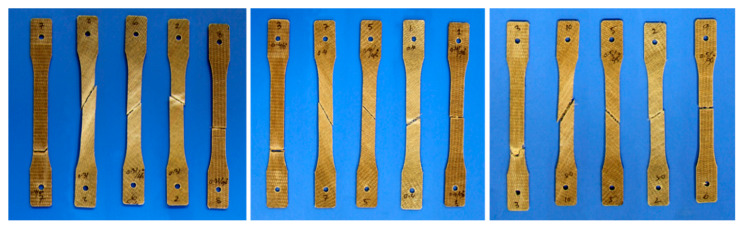
Tensile fracture samples of different fibre volume fractions and orientations. **Left** (*V_f_* = 0.31), **middle** (*V_f_* = 0.40), and **right** (*V_f_* = 0.50), and 0°, 30°, 45°, 60°, and 90° from left to right for each image.

Figure 18 shows examples of tensile fracture of flax/PP composite specimens. The specimens appear to be entirely detached into two pieces, except for some unbroken fibres between them. The samples with a 0° fibre orientation failed near the grip, whereas for other fibre orientations (30°, 45°, 60°, and 90°), the composite samples failed parallel to the fibre direction within the gauge length. The reason for failure near the grip (outside of the gauge length) is possibly due to the stresses generated by the gripping forces. However, tensile moduli for 0° fibre-oriented samples are reported as estimated in the strain range of 0.05 to 0.25%, which is considerably earlier than the failure strain (0.80 to 0.92%) of the samples (*θ* = 0°). The tensile strength for a 0° fibre orientation may not be accurate, as the samples failed outside the gauge length.

**Figure 19:**
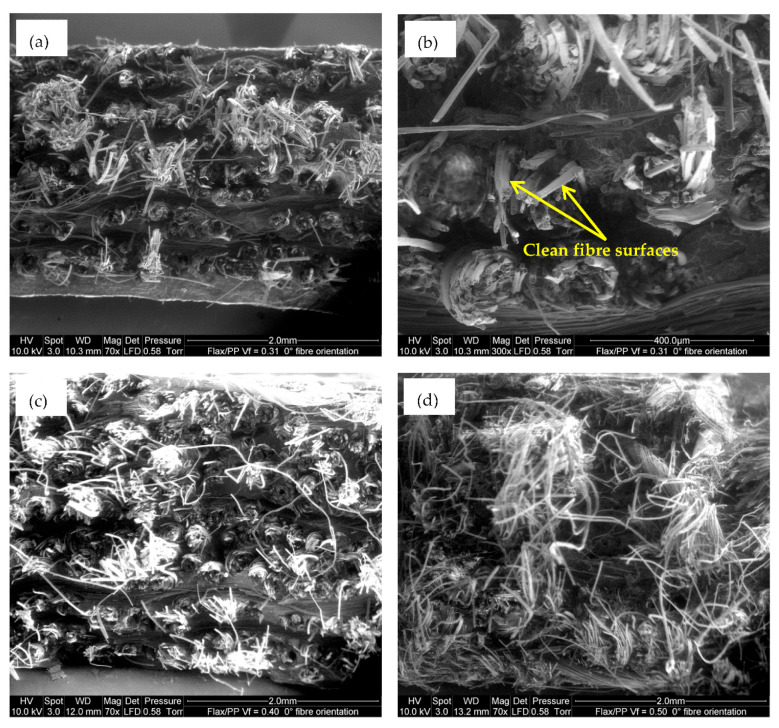
Tensile fracture surface images: (**a**,**b**) showing fibre pull-out for a fibre volume fraction and orientation of 0.31 and 0°, respectively, at two different magnifications, and (**c**,**d**) showing fibre pull-out for fibre volume fractions of 0.40 and 0.50, respectively, with a 0° fibre orientation.

As Figure 19a–d illustrate, fibre pull-out is the dominant failure mode, especially for samples with a 0° fibre orientation. It is difficult to see the matrix material, as fibre pull-out is much longer, and numerous fibre pull-outs (see Figure 19d) indicate even worse adhesion between flax fibres and PP in the case of fibre content of 50% than 31% by volume. The pulled-out fibres show that fibre surfaces are mostly free of the residual matrix, which is also evidence of poor adhesion between the fibres and matrix. The increased fibre content (i.e., *V_f_* = 0.50) may result in inadequate fibre wetting and non-uniform distribution of the fibre and matrix, lowering stress transfer between the fibre and matrix [85]. Moreover, the kink bands (see Figure 14b) may act as preferential sites for initiating cracks [86,87], negatively influencing the mechanical properties.

**Figure 20:**
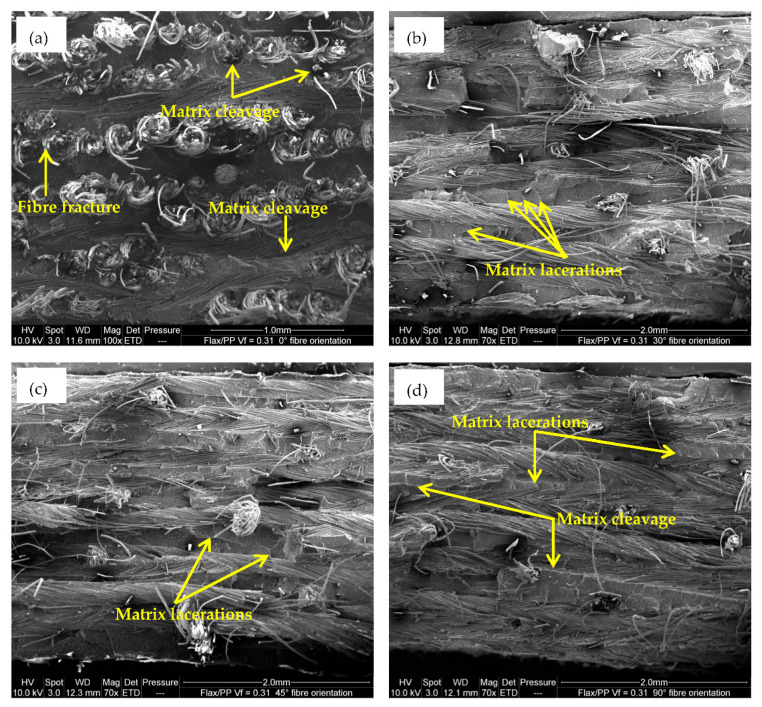
Tensile fracture surfaces of flax/PP composites with fibre orientations of (**a**) 0°, (**b**) 30°, (**c**) 45°, and (**d**) 90°.

The ESEM micrograph in Figure 20a also shows the serrated fracture and non-uniform surface because of fibre-dominated failure in the case of a 0° fibre-oriented sample. The matrix cleavage and fibres breakage at varying lengths are attributable to the longitudinal (i.e., 0°) tensile fracture of the composite [88].

When the fibre orientation is changed from 0° to off-axis angles (30°, 45°, 60°, and 90°), the samples fail easily along the fibre/matrix interfaces, as the interfaces are relatively weak. These are the consequences of fibre/matrix interfacial failure, matrix shear failure, and matrix tensile rupture [89]. Morey and Wool [90] pointed out that if the fibre/matrix interfacial strength is poor, failure occurs at the interface due to shear stresses. Other authors also reported that the interlaminar shear stresses and the transverse tensile stresses become more dominant as fibre orientation increases [91]. Figure 20b,c confirm the matrix lacerations, which indicate some shear fracture [88] for off-axis loading angles. Some matrix cleavage (see Figure 20d) is also observed because of the transverse tensile fracture of the matrix [88]. However, matrix lacerations are seen in matrix-rich zones, suggesting some shear fracture. Hence, while stress-strain curves show that composites with off-axis loading angles possess a ductile behaviour before fracture, the ESEM studies of tensile fracture samples indicate tensile failures occur due to debonding, fibre pull-out and breakage, weak fibre/matrix interface, and brittle fracture of the matrix.

#### 3.2.3. Tensile Properties of Flax/PP Composites: A Comparative Study

With plant fibre composites, there are large variations in the parameters (e.g., fibre volume fraction, fibre orientation, fibre aspect ratio, fibre stiffness, matrix type (thermoplastic or thermoset)), and manufacturing techniques (e.g., injection moulding, compression moulding, prepregging with autoclave consolidation, vacuum bagging, and resin transfer moulding). In addition, a number of variables, such as testing speed, gauge length, temperature, and moisture content, influence the measured properties, which are not often reported in the literature. These make comparisons of the manufactured flax/PP composites less straightforward to E-glass fibre and other plant fibre (i.e., short/long flax fibre) composites available in the literature. The comparisons are made based on the above considerations. These will be applicable throughout all comparisons of FFPCs with GFPCs and other plant fibre composites.

Table 2 compares the tensile properties of the manufactured flax/PP composites with those found in the literature for E-glass/PP and flax/PP composites. Unidirectional (UD) E-glass/PP composites (*θ* = 0°) outperform unidirectional flax/PP composites (*θ* = 0°) in terms of tensile stiffness and strength. The tensile modulus and strength of E-glass composites (*V_f_* = 0.35 and *θ* = 0°) are 26.5 GPa and 700 MPa, respectively, as reported by Gil [92]. These values are significantly higher than the tensile modulus (16.66 GPa) and strength (125.39 MPa), respectively, of flax/PP composites (*V_f_* = 0.31 and *θ* = 0). The superior properties of aligned GFPCs compared to aligned FFPCs are not only because of the higher properties of E-glass fibres compared to flax fibres (see Table 3) but also because of the differences in their structures. Aligned GFPCs employ continuous fibres with almost perfect alignment, whereas FFPCs use yarns with discontinuous fibers that are twisted to some degree. However, an advantage of flax fibres in comparison to E-glass fibres is that their density is lower (about half (≈55%) lower) (see Table 3), and thus, plant fibre composites are 30–40% lighter than GFPCs at the same fibre content [93]. Hence, the specific properties of the composites are important; especially, specific tensile modulus and strength are often taken into consideration in the material selection process for structures subjected to tensile load [94].

The specific tensile modulus and strength of E-glass fibre composites (*V_f_* = 0.35 and *θ* = 0°) are 17.43 GPa/gcm^−3^ and 461 MPa/gcm^−3^, respectively. On the other hand, flax/PP composites (*V_f_* = 0.31 and *θ* = 0°) demonstrate the specific tensile modulus and strength of 15.72 GPa/gcm^−3^ and 118.29 MPa/gcm^−3^, respectively (noted that there are differences in fibre volume fraction and manufacturing technique). This demonstrates that the specific tensile modulus of aligned flax fibre composites can be equivalent to E-glass fibre composites, but the specific tensile strength is considerably lower (≈26%) than that of E-glass. The poor specific tensile strength could be because of the lower strength of flax fibres compared to glass fibres, as presented in Table 3. However, the specific stiffness of flax fibre composites clearly demonstrates that flax has the potential to be employed in load-bearing structures and as a prospective alternative to E-glass in stiffness-critical applications. Flax fibres (i.e., lighter than E-glass fibres) may also provide a lightweight and stiff alternative to E-glass fibres at the same fibre loading.

It can also be pointed out that the specific tensile strength (118–150 MPa/gcm^−3^) of flax fibre composites (*θ* = 0°) is higher than E-glass mat composites (≈24 MPa/gcm^−3^). Even for 30° fibre orientation (*V_f_* = 0.31), the specific strength (28.10 MPa/gcm^−3^) of flax fibre composite is better than E-glass mat composites.

The tensile modulus and strength of flax/PP samples (*V_f_* = 0.43 and *θ* = 0°), as shown by Madsen and Lilholt [95], outperform the studied samples with a fibre volume fraction of 0.50 (*θ* = 0°). The possible reasons for the lower value obtained in this study compared to the literature are: (1) the former was manufactured using compression moulding, which is a better manufacturing technique than vacuum bagging. This is because there is more void content in the samples manufactured by vacuum bagging [70]. (2) The properties can vary a lot depending on the natural origin of fibres, which implies an inherently large variation in fibre properties. The textile monofilament flax yarn (density of 1.56 gcm^−3^, supplied by Linificio E Canapificio Nazionale, Italy) was used for the former; on the other hand, flax fabric (density of 1.42 gcm^−3^, supplied by Lineo, Belgium) was used in this study. Furthermore, for the former, the samples were humidity-conditioned. Some variation may also be added by the processing of fibres, structural morphology, and their chemical composition.

The tensile properties of UD FFPCs (*V_f_* = 0.40 and *θ* = 0°) are about 2.5 times higher (this study) than short randomly-oriented flax fibre composites (*V_f_* = 0.40), as observed in reference [99]. This is due to high fibre aspect ratios and good fibre alignment, enhancing the degree of reinforcement efficiency, although the flax fibres are twisted to some degree within the yarns (see Figure 6).

#### 3.2.4. Flexural Properties

**Figure 21:**
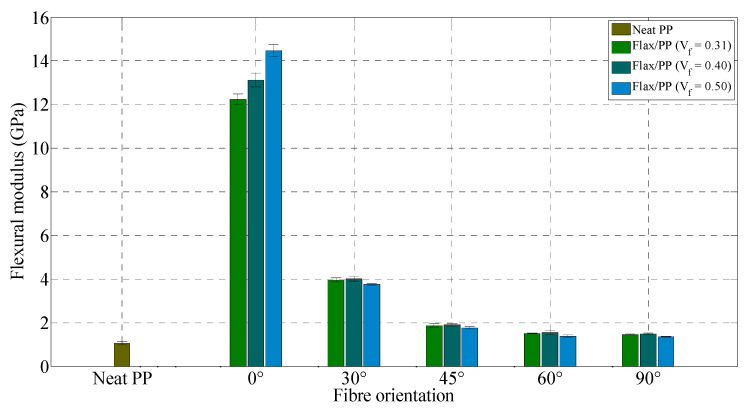
Flexural modulus for neat PP and flax/PP samples of different fibre volume fractions and orientations.

**Figure 22:**
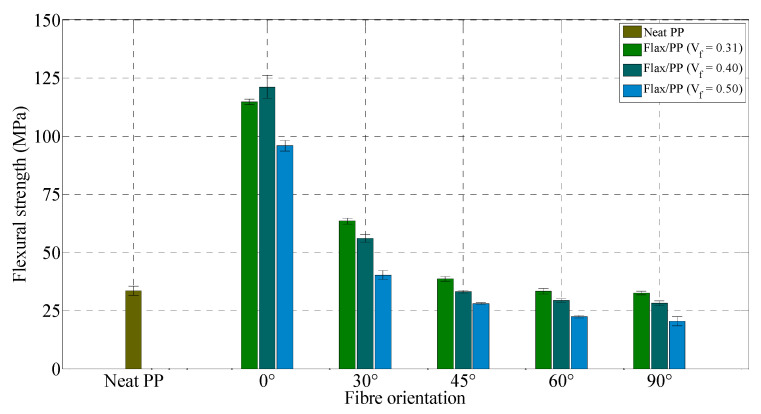
Flexural strength for neat PP and flax/PP samples of different fibre volume fractions and orientations.

Figure 21 and Figure 22 illustrate the flexural properties of neat PP and flax/PP samples containing different fibre contents and orientations. As the orientation of fibres is varied from 0° to 90°, flexural properties of the composite samples demonstrate a decreasing trend in their moduli and strengths like the tensile properties. The flexural moduli are found to be higher for composites with a fibre volume fraction of 0.40 than those of other fibre volume fractions’ composites, apart from 0° fibre orientation. This inconsistency may be because all fibres are able to contribute to the composite modulus during the flexural loading in the case of 0° fibre-oriented samples. Flexural properties of composites, like tensile properties, drop significantly with increasing angles between 0° and 30°. However, the flexural moduli do not exhibit large variations between 45° and 90°. The lowest flexural properties are measured for the samples with a 90° fibre orientation, similar to the previous findings of the lowest tensile properties at an angle of 90°.

Generally, adding fibres increases the flexural moduli of composites, and the 0° fibre-oriented sample shows the highest increase in its properties. With the addition of 40% flax fibres by volume (*θ* = 0°), the flexural modulus and strength are raised by 1126% (from 1.07 GPa to 13.12 GPa) and 243% (from 33.50 MPa to 114.80 MPa), respectively, over the neat PP sample. This reveals an increase in rigidity of the neat matrix material with the incorporation of fibres. The embedding of more fibres into PP does not have a significant effect on the flexural moduli and strengths, particularly for the samples with fibre orientations of 30°, 45°, 60°, and 90°, which is a sign of poor adhesion between flax fibres and PP. As a result, the stress could not transfer effectively from the PP to the stronger fibres. There is a strong dependence between the impregnation quality and the mechanical properties of the material. It is seen that the impregnation of flax fibres by PP is not good as fibres are as yarns, and effects are more severe when more fibres are added. Consequently, increasing flax fibres does not contribute to raising the flexural moduli or strengths. Notably, the standard deviations of flexural modulus and strength of five replicated specimens of flax composite range from 0.02 to 0.65 GPa and 0.09 to 6.34 MPa, respectively, regardless of fibre contents and orientations. Most measurements have small standard deviations, indicating measurement consistency.

**Figure 23:**
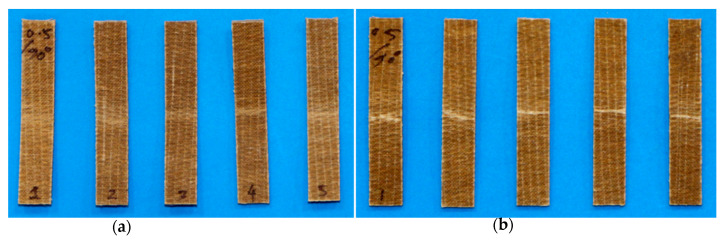
Typical flexural fracture samples: (**a**) Side of the applied load and (**b**) opposite side of the applied load.

Figure 23a,b show the compression and tensile sides, respectively, of the flexural fracture samples. There is no significant damage except the curvature of all composite samples.

#### 3.2.5. Flexural Properties of Flax/PP Composites: A Comparative Study

Table 4 compares the flexural properties of the manufactured flax/PP composites with E-glass/PP and flax/PP composites available in the literature. The specific flexural modulus and strength of the E-glass/PP composite (*V_f_* = 0.35 and *θ* = 0°) are 15.87 GPa/gcm^−3^ and 276.67 MPa/gcm^−3^, respectively [99]. On the other hand, in this study, flax/PP composite (*V_f_* = 0.31 and *θ* = 0°) shows the specific flexural modulus and strength of 11.55 GPa/gcm^−3^ and 90.47 MPa/gcm^−3^, respectively. The specific modulus and strength of the flax/PP composite are 73% and 33% of the modulus and strength, respectively, of the E-glass/PP composite (notably, the differences are in the fibre volume fraction and manufacturing technique). This indicates that the flexural strength of flax/PP composites cannot compare well with that of E-glass/PP, as similar to the tensile strength. Again, this is probably because of the comparatively lower mechanical strength of flax fibres in comparison to glass fibres (see Table 3). However, the specific flexural modulus and strength of aligned flax/PP composite (*V_f_* = 0.31 and *θ* = 0°) are 3 and 2.1 times higher than the specific modulus (3.84 GPa/gcm^−3^) and strength (42.55 MPa/gcm^−3^), respectively, of glass fibre mat composite (*V_f_* = 0.30). Compared to randomly-oriented short flax fibre reinforced composite (*V_f_* = 0.40) [93], UD flax/PP composite (*V_f_* = 0.40 and *θ* = 0°) demonstrates improvements of 93% and 48% in flexural modulus and strength, respectively.

Overall, the tensile and flexural moduli increase up to a fibre content of 40% by volume, and then it is found to decrease with the addition of more fibres, while tensile and flexural strengths decrease with increasing fibre volume fraction when compared to the composites with a fibre volume fraction of 0.31. The specimens with a 0° fibre orientation have the maximum tensile and flexural modulus and strength compared to other fibre orientations. The tensile strain at break is found to be higher for a 30° fibre orientation than other fibre orientations. Unidirectional flax/PP composites (*θ* = 0°) may have similar specific stiffness to unidirectional glass fibre composites (*θ* = 0°), but their specific strength is low.

The off-axial composite samples show crack propagation along the fibre direction in addition to shear failure at fibre/matrix interface under tension testing. The damaged samples experience matrix- and interface-dominated brittle failures and ductile failure. In particular, composite samples with a fibre orientation of 0° show a brittle failure, whereas composites with fibre orientations of 30°, 45°, 60°, and 90° show ductile behaviour before fracture. The ESEM analysis confirms poor fibre/matrix adhesion (attributed to poor wetting of the fibres in the matrix) and failure of specimens due to fibre debonding, fibre pull-out and breakage, matrix cracking, and inadequate fibre/matrix adhesion. However, the fracture samples exhibit no evidence of substantial failure under three-point bending measurements.

## 4. Concluding Remarks

The dynamic behavior of FFPCs was examined using DMA up to frequencies of 100 Hz. The effect of the fibre content, fibre orientation, and frequency was estimated from vibration measurements to determine the parameters that significantly influence damping in FFPCs. Introducing fibre content produces a substantial rise in storage modulus. A significant property improvement is observed for the composite with a 40% flax fibre by volume. As the frequency increases, the storage modulus increases in the range of 4–10%, whereas the loss factor rises by 14–28%. The loss factor is typically in the range of 4–5.5%, regardless of fibre orientation, volume fraction, and frequency. However, flax/PP composites with a fiber volume fraction of 0.40 (*θ* = 45°) achieve a good trade-off between stiffness and damping. Higher damping (i.e., an increase of 281 to 953%) can be obtained with flax fibre/PP composites compared to more common composite materials, such as glass fibre/epoxy composites.

The variation in fibre orientation affects mechanical properties. The highest tensile modulus and strength are found in the case of 0° fibre orientation. The aligned composite specimens (*θ* = 0°) have tensile modulus and strength in the range of 17–21 GPa and 125–173 MPa, respectively, for fibre volume fractions of 0.31–0.50, while for the same fibre volume fraction range, composites with a 0° fibre orientation have flexural modulus and strength of 12–15 GPa and 96–121 MPa, respectively. These properties suggest that FFPCs are contenders as substitutes for conventional materials in structural applications. The tensile strain at break is found to be higher for a 30° fibre orientation than for other fibre orientations. Unidirectional flax/PP composites (*θ* = 0°) can provide comparable specific stiffness to unidirectional glass fibre composites (*θ* = 0°), but the specific strength of flax/PP is poor. 

Some parameters, such as fibre content and fibre orientation on FFPCs, were varied, and it was found that increasing the fibre orientation is always beneficial to some properties (e.g., damping), but it is detrimental to other properties (e.g., stiffness). However, the sample with a fibre volume fraction of 0.40 and orientation of 45° is found to give a good balance of properties.

Morphological investigation reveals that the interfacial adhesion between the flax fibres and PP matrix decreases as more fibres are added. In addition, the yarns (i.e., fibre bundles) are believed to act more like an obstacle for impregnation during manufacturing and thus result in fibre-to-fibre contact, reducing the mechanical properties (tensile and flexural) of the resulting polymer composites. In tension, the FFPCs show a typical brittle fracture mode. The ESEM study clearly reveals that the tensile failures of FFPCs occur due to debonding, fibre pull-out and breakage, weak fibre/matrix interface (attributed to poor wetting of the fibres in the matrix), and brittle fracture of the matrix.

Overall, it may be inferred that the high damping capability of flax fibre-reinforced composite materials with high stiffness and low weight make them appropriate for applications where high stiffness and high damping are sought. Potential applications may be considered in areas, for example, automobiles, aircraft interiors, machinery parts, sporting goods (to increase control and comfort), and musical instruments. The benefits of having high loss factors with lightweight FFPCs are prolonged service life of the composite parts, reduction in noise and vibration, reduction in the effect of dynamic loading on the structural response, and a decrease in weight. Moreover, using plant-based fibres in composites minimises greenhouse gas emissions, lessens dependence on non-renewable petroleum-based resources, and reduces environmental impacts due to their biodegradability, making them a more sustainable option for various applications.

## Figures and Tables

**Table 1:** Number of layers of PP and flax fabric, nominal fibre weight fractions, fibre volume fractions, and thicknesses of composite panels.

Layers of PP	Layers of Flax	Nominal Fibre Weight Fraction, *W_f_*	Nominal Fibre Volume Fraction, *V_f_*	Nominal Thickness (mm)
6	8	0.40	0.31	3.03
5	10	0.50	0.40	3.00
4	12	0.60	0.50	3.28

**Table 2:** A comparison of tensile properties of the manufactured flax/PP composites with E-glass/PP and flax/PP composites found in the literature.

Manufacturing Technique	Composite	Fibre Orientation	Fibre Content ^a^ (%)	Density (gcm^−3^)	Tensile Modulus (GPa)	Specific Tensile Modulus (GPa/gcm^−3^)	Tensile Strength (MPa)	Specific Tensile Strength (MPa/gcm^−3^)	Reference
Vacuum bagging	Flax/PP	0°	31 v ≈ 40 wt	1.06	16.66	15.72	125.39	118.29	This study
	Flax/PP	0°	40 v ≈ 50 wt	1.10	19.99	18.17	144.76	131.60	This study
	Flax/PP	0°	50 v ≈ 60 wt	1.15	20.44	17.77	173.08	150.50	This study
	Flax/PP	30°–90°	31–50 v	1.06–1.15	1.12–3.45	0.97–3.17	15.53–29.78	13.50–28.09	This study
Compression moulding	Flax/PP	0°	43 v	1.14	26.90	23.60	251.10	220.26	[24]
Compression moulding	Flax/PP	Random	40 v	1.10	8.80	8	57	52	[93]
Injection moulding	Flax/PP	Random	40 wt	1.08–1.16	1.86–2.43	1.72–2.15	20.67–34.70	19.14–30.36	[95]
Compression moulding	E-glass/PP	0°	35 v	1.52	26.5	17.43	700	461	[92]
Compression moulding	E-glass/PP	CSM ^b^	30 v	1.41	7.02	4.98	33.74	23.93	[96]

^a^ Note that the specific properties have been calculated using the composite density $\left(\rho_{c}=\rho_{f}V_{f}+\rho_{m}V_{m}\right)$ if fibre volume fraction $\left(V_{f}\right)$ is mentioned in the literature or $\rho_{c}=(\rho_{f}\rho_{m})/(\rho_{f}+W_{f}(\rho_{m}-\rho_{f}))$ if fibre weight fraction $\left(W_{f}\right)$ is mentioned in the literature. ^b^ CSM = Chopped strand mat.

**Table 3:** Mechanical properties of flax and E-glass fibres [97,98].

Fibre	Density (gcm^−3^)	Stiffness (GPa)	Tensile Strength (MPa)	Specific Stiffness (GPa/gcm^−3^)	Specific Tensile Strength (MPa/gcm^−3^)	Failure Strain (%)
Flax	1.4–1.5	55–75	800–1500	38–52	550–1030	1.2–3.3
E-glass	2.5–2.59	70–74	2000–2400	27–29	780–940	1.8–4.8

**Table 4:** A comparison of flexural properties of the manufactured flax/PP composites with E-glass/PP and flax/PP composites found in the literature.

Manufacturing Technique	Composite	Fibre Orientation	Fibre Content ^a^ (%)	Density (gcm^−3^)	Flexural Modulus (GPa)	Specific Flexural Modulus (GPa/gcm^−3^)	Flexural Strength (MPa)	Specific Flexural Strength (MPa/gcm^−3^)	Reference
Vacuum bagging	Flax/PP	0°	31 v ≈ 40 wt	1.06	12.24	11.55	95.90	90.47	This study
	Flax/PP	0°	40 v ≈ 50 wt	1.10	13.12	11.93	114.80	104.36	This study
	Flax/PP	0°	50 v ≈ 60 wt	1.15	14.48	12.59	121.10	105.30	This study
	Flax/PP	30°–90°	31–50 v	1.06–1.15	1.07–3.96	0.93–3.74	20.4–63.50	17.74–59.91	This study
Compression moulding	Flax/MAA-PP ^b^	0°	21.6 v	1.02	10.75	10.54	122.50	120.10	[100]
Compression moulding	Flax/PP	Random	40 v	1.10	6.80	6.18	77.65	70.59	[93]
Injection moulding	Flax/PP	Random	40–60 wt	1.08	5.50–8.22	5.09–7.08	38.82–43.52	33.47–4.30	[95]
Compression moulding	E-glass/PP	0°	35 v	1.50	23.8	15.87	415	276.67	[99]
Compression moulding	E-glass/PP	CSM ^c^	30 v	1.41	5.41	3.84	60.00	42.55	[96]

^a^ Noted that the specific properties have been calculated using the composite density $\left(\rho_{c}=\rho_{f}V_{f}+\rho_{m}V_{m}\right)$ if fibre volume fraction $\left(V_{f}\right)$ is mentioned in the literature or $\rho_{c}=(\rho_{f}\rho_{m})/(\rho_{f}+W_{f}(\rho_{m}-\rho_{f}))$ if fibre weight fraction $\left(W_{f}\right)$ is mentioned in the literature. ^b^ MAA-PP = Maleic acid anhydride modified polypropylene, ^c^ CSM = Chopped strand mat.

## Data Availability

All data of this manuscript are fully available.
